# Role of meningeal lymphatic vessels in brain homeostasis

**DOI:** 10.3389/fimmu.2025.1593630

**Published:** 2025-06-19

**Authors:** Meng-Ying Zhao, Chao-Yuan Ye, Yuan-Cheng Liu, Xiao-Ming Wang, Jun-Cai Fu, Xin-Yuan Liu, Rui Zhu, Yi-Zhao Li, Qing Tian

**Affiliations:** Department of Pathophysiology, School of Basic Medicine, Key Laboratory of Neurological Diseases of Hubei Province and National Education Ministry, Tongji Medical College, Huazhong University of Science and Technology, Wuhan, China

**Keywords:** meningeal lymphatic vessels, brain homeostasis, cerebrospinal fluid drainage, neuroinflammation, neurological disorders, imaging technique

## Abstract

Meningeal lymphatic vessels (MLVs) form an important bridging structure between the brain and periphery, which drains cerebral metabolites in cerebrospinal fluid (CSF) and brain antigens to deep cervical lymph nodes (dCLNs), to maintain brain homeostasis. Increasing evidence reveals the importance of MLVs in brain ageing and various central nervous system (CNS) diseases, such as Alzheimer’s disease (AD), Parkinson’s disease (PD), traumatic brain injury (TBI), and multiple sclerosis (MS). Advances in research techniques have provided detailed insights into the structure and functions of MLVs, highlighting the therapeutic potential of targeting MLVs for related diseases. Here, we perform a systematic review of the features and functional regulation of MLVs, their associations with brain disorders, as well as some methodological advances in imaging of MLVs and the drainage pathway.

## Background

The blood-brain barrier (BBB), a highly selective structure, isolates the CNS from peripheral systems while hindering substance exchange between the periphery and CNS (1). Accordingly, the homeostasis of the brain is traditionally thought to depend mainly on stations of neurovascular units (NVUs), which involve neurons, astrocytes, capillary-associated microglia, pericytes, endothelial cells and perivascular macrophages, and the signal communications among them (1, 2). A growing number of studies shedding light on the interactions between the brain and periphery, such as peripheral organs, peripheral metabolic system, peripheral immune system and gut microbiota, have indicated that the homeostasis of brain is closely related to the overall state of the body, underscoring the importance of communication pathways between the brain and periphery. Recently, MLVs have been identified as important bridging structures between the periphery and CNS, which drains CSF, metabolic waste, and immune cells that carry antigens from brain towards dCLNs (3, 4), revolutionizing our understanding of brain homeostasis.

MLVs were first named by Antoine Louveau and colleagues. They demonstrated the presence of a lymphatic vascular system around the dural sinuses of mouse meninges, marked by the expression of the lymphatic endothelial hyaluronic acid receptor-1 (LYVE-1), a lymphatic endothelial cell (LEC) marker (3). In fact, as early as the late 18th century, an Italian anatomist named Paolo Mascagni first mentioned the possible existence of meningeal lymphatic tissue in the dura, but it remained unrecognized by the scientific mainstream (5). In the following two centuries, owing to the limitations of experimental techniques, scientists did not make substantial progress in exploring the meningeal lymphatic system. In 2015, Antoine Louveau and Aleksanteri Aspelund et al. confirmed the existence of MLVs and discovered their role in transporting macromolecules in the brain to dCLNs (3, 6). Subsequently in 2017, the localization of MLVs in zebrafish (7), rats (8), humans and nonhuman primates (9) was respectively reported.

MLVs can participate in maintaining brain homeostasis from two aspects: substances drainage and immunity. On the one hand, MLVs can remove metabolic wastes from the brain to the periphery and reduce the deposition of harmful substances in CNS (3, 10). On the other hand, MLVs can transport brain antigens (4), and there are many immune cells (including the antigen presenting cells and T cells) on the dura mater for immune surveillance of the CNS (11). In addition, impaired MLVs can also reshape the morphology and function of microglia (12, 13). In 2016, Antoine Louveau et al. proposed that strategies for promoting meningeal lymphatic drainage may be beneficial for some neurological disorders, which opened the prelude to the study of MLVs in CNS diseases (14). In this review, we aim to summarize the relevant characteristics of MLVs, the pathway for CSF clearance and immune cell trafficking, the functional regulation of MLVs and their connection with some neurological diseases, as well as several related imaging methods.

## Location of MLVs

**Figure 1:**
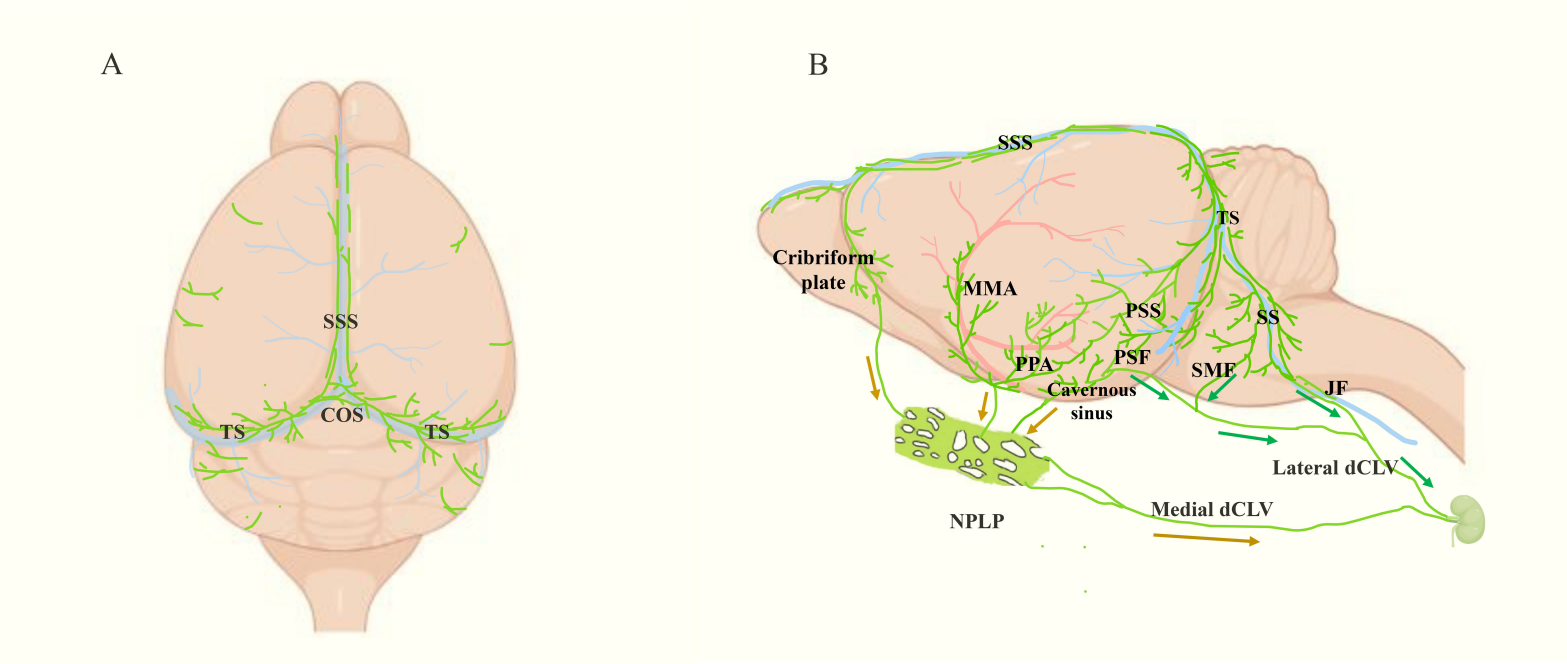
Localization and drainage pathway of mouse MLVs (green). **(A)** Top view of MLVs, which mainly shows the dorsal MLVs located along SSS and TS. SSS: superior sagittal sinus, TS, transverse sinus. **(B)** Side view of the meningeal lymphatic system, including the basal MLVs located near the PSS, SS, MMA and PPA. Besides, the drainage pathways from CNS to dCLN are shown following the arrows. Among them, the green arrows point to the direction of basal MLVs leaving the intracranial cavity. They get out of the skull at PSF, JF and SMF, then reach the dCLN via the lateral deep cervical lymph vessels (dCLV). The brown arrows indicate the lymphatic drainage pathway of the three upstream regions of nasopharyngeal lymphatic plexus (NPLP), which finally arrive at dCLN via medial dCLV. PSS, petrosquamosal sinus; SS, sigmoid sinus; MMA, middle meningeal artery; PPA, pterygopalatine artery; PSF, petrosquamous fissure; JF, jugular foramen; SMF, stylomastoid foramina.

**Figure 2:**
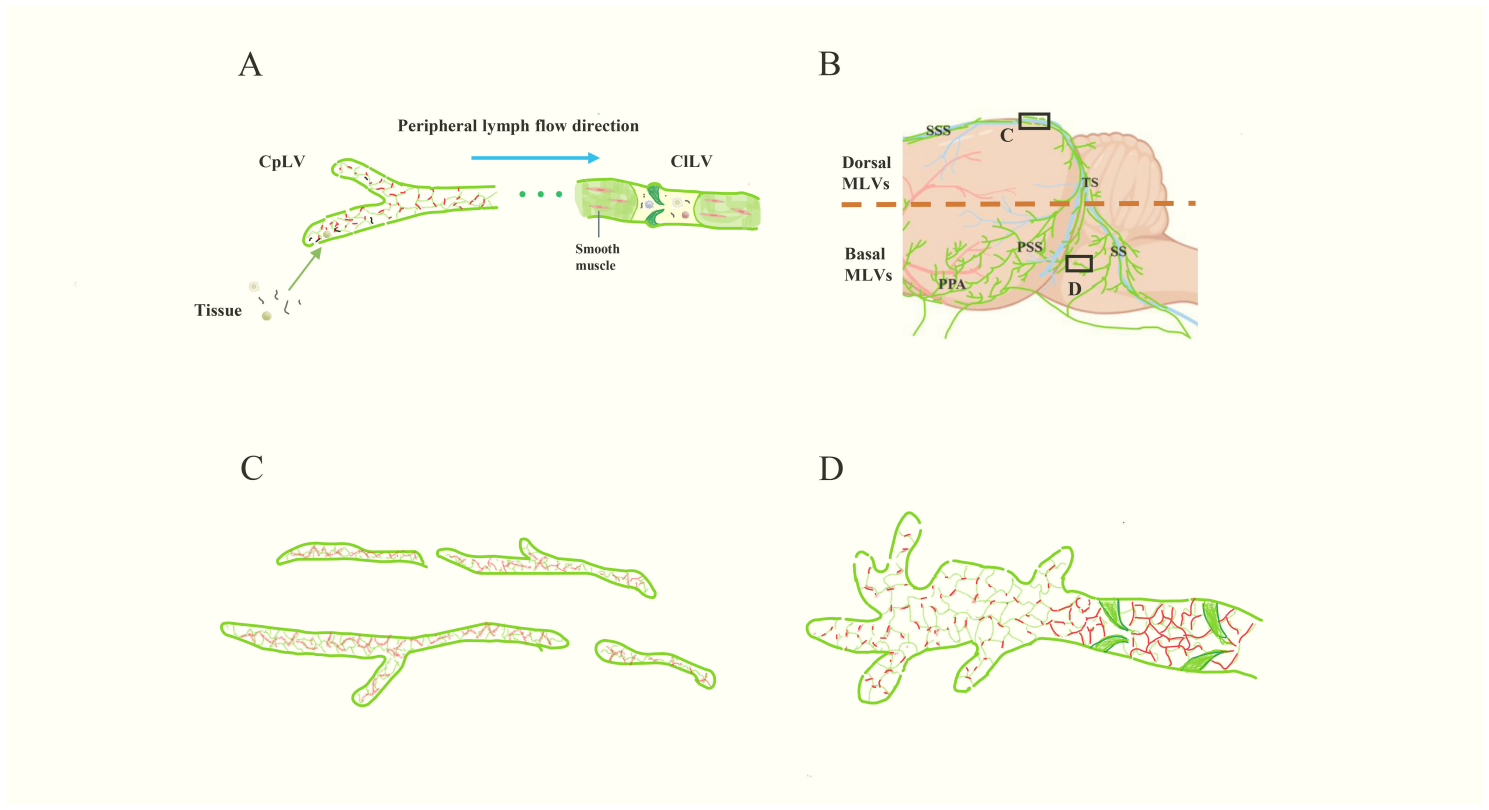
The structure of peripheral lymphatic vessels and MLVs. **(A)** The schematic diagram of peripheral lymphatic vessels. The left are CpLVs, which have loosely arranged endothelium like buttons (red) and drain ISF and large molecules. Then the lymph inside flows into ClLVs, which have tightly arranged endothelium, smooth muscle and lymphatic valves. **(B)** The schematic image of MLVs (green). The lymphatic vessels in upper part are dorsal MLVs and the lymphatic vessels in the lower part are basal MLVs. **(C, D)** are the magnifications of the images in the black boxes in **(B)**. **(C)** represents the dorsal MLVs, which have few branches, tightly arranged endothelium like zipper (red), discontinuous structure and small diameter. **(D)** represents the basal MLVs with large lumens, lymphatic valves, abundant branches, and loosely arranged endothelium, which is conducive to CSF drainage. Besides, there are lymphatic valves at the proximal part and the endothelium is closely arranged like the ClLVs.

In studies of the peripheral lymphatic system, markers of LECs, such as LYVE-1 and prospero homeobox protein-1 (Prox-1), are often used to show the location and morphological structure of lymphatic vessels (15–17). Additionally, these markers are found in MLVs specifically in the brain. Immunofluorescence staining of these markers combined with high-resolution imaging technologies helped to identify the presence of MLVs (3, 6). Based on the location, MLVs can be mainly categorized into dorsal MLVs and basal MLVs. The dorsal MLVs are situated near the superior sagittal sinus (SSS) and transverse sinus (TS), wrapped in the dura and far from the subarachnoid space (**Figure 1A**) (3, 6, 18). The basal MLVs are located near the petrosquamosal sinus (PSS) and sigmoid sinus (SS), along the middle meningeal artery (MMA), pterygopalatine artery (PPA), and vein of Galen, and close to the subarachnoid space (**Figure 1B**) (3, 6, 18). Although the positioning is different, the basal MLVs and the dorsal MLVs are connected and eventually leave the CNS from the base of the skull (**Figures 1B**, **2B**). In addition, there are some MLVs distributing near the cribriform plate, cavernous sinus and pituitary gland (6, 18). In the eyes and vertebrae, there are also some lymphatic vessels similar to MLVs (19, 20). The anterior and posterior chambers of the eyes have distinct lymphatic drainage systems, while the latter can drain to the dCLNs through lymphatics in the optic nerve sheath (19). Vertebral lymphatic vessels (VLVs) are mainly distributed in epidural space and arranged in segments along the vertebrae, forming a loop between each vertebra and connecting to peripheral lymphatics through the ligamentum flavum, dorsal facet joint and intervertebral foramen (20).

**Figure 3:**
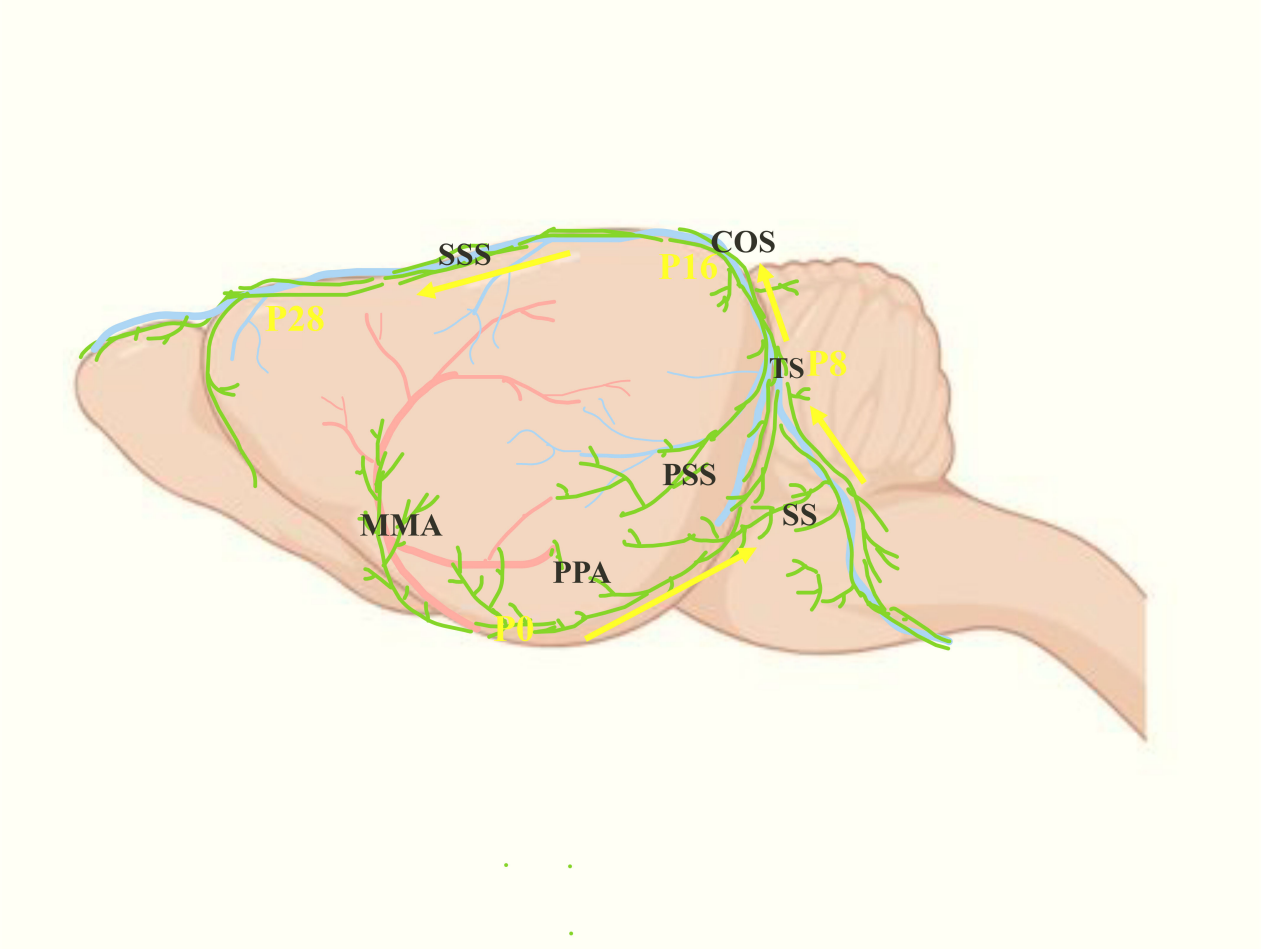
Postnatal development progress of the MLVs (green). The yellow arrows represent the development direction of MLVs after birth: from MMA and PPA to SS, then to TS, and finally to SSS after intersecting at COS.

Before birth, the initial MLVs in mice are located near the foramen magnum and PPA. After birth, MLVs begin to develop along the MMA and PPA, grow along the dorsolateral side of SS, and reach the TS at the postnatal day 8 (P8). Following that, MLVs congregate at the confluence of the sinuses (COS) along the bilateral TS at P16, then extend along the SSS, and reach the rostral end of the SSS at P28, completing the basic development process (**Figure 3**) (21, 22).

## Structures of MLVs

In the periphery, lymphatic vessels are divided into capillary lymphatic vessels (CpLVs) and collecting lymphatic vessels (ClLVs), with distinct structural features (**Figure 2A**) (17, 23). CpLVs have loosely arranged endothelium resembling buttons, which is conducive to draining interstitial fluid (ISF) and absorbing large molecules such as proteins. Anchoring filaments, another feature of initial lymphatic vessels, connect the membrane of LECs to surrounding elastic fibers, allowing CpLVs to remain open and absorb ISF (23, 24). CpLVs lack valves and smooth muscle, so lymphatic fluid can flow freely in all directions within the initial lymphatic plexus, whereas ClLVs transport lymphatic fluid from CpLVs to the lymph nodes and lymphatic trunks(**Figure 2A**) (17, 23, 25). The compact wall structure of ClLVs is divided into three layers: endothelial, smooth muscle and collagen tissue. ClLVs, like veins, have valves that permit fluid to flow in only one direction (proximal) (26). Through the functioning of smooth muscle, ClLVs undergo autonomous contractions to periodically propel lymph flow (26, 27).

MLVs present some characteristics consistent with initial lymphatic vessels, including the absence of smooth muscle and autonomous contractions (3, 6), leaving the driving force behind lymph flow in MLVs an enigma. The dorsal MLVs and basal MLVs are connected, but exhibit different structural characteristics(**Figures 2B-D**). The dorsal MLVs are small in diameter, discontinuous in structure, with few branches, and do not express integrin α-9, which is a marker of the lymphatic valve (**Figure 2C**) (28). Different from the directionless nature of CpLVs, the lymph in dorsal MLVs flows towards the cribriform plate in human (29). When Antoine Louveau et al. discovered MLVs in 2015, they proposed that Claudin-5 and vascular endothelial-cadherin (VE-Cadherin) have point-like distributions on dorsal MLVs (3). However, in 2019, Ji Hoon Ahn et al. reported that VE-Cadherin shows a continuous and closed distribution in dorsal MLVs, and the endothelial cells are closely connected like zippers, which are unsuitable for CSF drainage (**Figure 2C**) (18). In contrast, basal MLVs have large lumens, abundant branches, and the endothelial cells are leaf-like, loosely arranged like buttons, combining the characteristics of CpLVs and ClLVs (**Figure 2D**). However, at the proximal part, the endothelium is closely arranged like ClLVs and the lymphatic valves lead to the main flow direction of MLVs being intracranial to dCLNs (**Figure 2D**) (18). These structures make basal MLVs the main bearers of meningeal lymphatic drainage. However, the existence of anchoring filaments and layered structure in MLVs has not been reported so far, and how MLVs remain open and absorb CSF is still a question to be explored.

## Drainage pathway through MLVs

At the beginning of the discovery of MLVs, researchers also revealed their function of draining CSF to dCLNs (3, 6). Initially, they were thought to get out of the skull following the cranial nerves, which were called perineural routes, including: along the olfactory nerve (near the cribriform plate), the trigeminal nerve and optic nerve (near the orbit), the facial nerve (through the stylomastoid foramina, SMF), as well as the glossopharyngeal, vagus, and accessory nerves (through the jugular foramen, JF) (6, 30). However, in 2019, Ji Hoon Ahn and colleagues discovered that MLVs remained intact after cranial nerve removal, suggesting that MLVs are not part of the perineuronal lymphatic system and may be simply concomitant with nerves (18). They observed basal MLVs draining CSF tracers from the petrosquamous fissure (PSF), JF, and SMF to periphery, connecting with extracranial lymphatics (lateral deep cervical lymph vessels, lateral dCLVs) and finally arriving to the dCLNs (**Figure 1B**) (18). In 2024, Jin-Hui Yoon et al. found that the nasopharyngeal lymphatic plexus (NPLP) is also a crucial hub for meningeal lymphatic drainage. The NPLP shares structural characteristics with basal MLVs, featuring both capillary and collecting lymphatic vessel traits: lacking smooth muscle coverage but having valves and a mixture of button-like and zipper-like junctions (31). There are three lymphatic drainage regions upstream of NPLP. Region 1 includes the lymphatics near the pituitary gland and cavernous sinus. Region 2 encompasses the lymphatics in the anterior region of the basolateral dura near the MMA and PSS, which flow along the PPA. Region 3 includes the lymphatics near the cribriform plate. The downstream of NPLP is connected to the dCLNs by the medial dCLVs (**Figure 1B**) (31).

However, at the starting point of meningeal lymphatic drainage pathways, how does CSF from the subarachnoid space access dural lymphatic vessels? This longstanding question was resolved in 2024 with the discovery of the arachnoid cuff exit (ACE) by Leon C. D. Smyth and colleagues, which provides a new perspective for CSF discharge other than arachnoid granules (32). In mouse models, they discovered that bridging veins traversing from the subarachnoid space into dural sinus are surrounded by cuff-like structures which are encased by arachnoid barrier cells and dural border cells, as well as a variety of immune cells. They termed the structure ACE points, where the phenotype of endothelial cells changes from that of the BBB (with specialized tight junctions) to that of peripheral blood vessels(with looser connection), providing an interface for molecular exchange (32, 33). Macromolecules from CSF were observed to migrate through ACE points into the dura mater, subsequently accessing meningeal lymphatic vessels. In human, magnetic resonance imaging (MRI) tracers transit along bridging veins in a similar manner (32). For immune cells aggregated near the dural sinus (11), the ACE point may also allow them to directly enter the subarachnoid space, realizing barrier-free trafficking of immune cells under neuroinflammatory conditions (32).

Traditionally, the meningeal lymphatic pathway has been usually described as an unidirectional channel from CNS to dCLNs. However, a study in 2024 revealed the bidirectional nature of lymphatic flow between CNS and dCLNs. Héctor M Ramos-Zaldívar et al. injected some nanoparticles, including superparamagnetic iron oxide, exosomes, gold nanorods, and Chinese ink nanoparticles, into dCLNs. Using MRI and histological analysis, they detected these nanoparticles in the brain, proving the reverse transport function of meningeal lymphatic pathway (34). This finding indicates that MLVs could serve as a novel route for delivering drugs to the brain, bypassing the BBB. In particular, they confirmed that exosomes can be transported backwards into CNS (34), which is a promising drug vehicle with perfect biocompatibility (35). Indeed, emerging evidence indicates that a similar lipid carrier named α-phospholipid nanoprobe (α-PLNPs) can carry drugs to prevent intracranial tumors via meningeal lymphatic reverse pathway (36).

The glymphatic system, another lymphatic system in CNS, is inextricably linked to MLVs, especially in terms of function. Serving as the bridge between CSF and brain ISF (33, 37), the glymphatic system can be considered as the upstream of meningeal lymphatic draining cerebral metabolites. Glymphatic system exchanges the solute between CSF and ISF, driven by cerebral arterial pulsation (38). Some cerebral metabolites and pathological molecule, like amyloid β (Aβ) and microtubule-associated protein (tau), can be cleared into CSF in order to be drained to periphery by MLVs, which function depends on the water channel aquaporin-4 (AQP4) located in the end feet of astrocytes (37–40). Enhancing the glymphatic system facilitates the discharge of molecules in CNS via meningeal lymphatic and may serve as a target for CNS waste removal (41, 42). Exploring the detailed connection between these two lymphatic systems in CNS is significant for clarifying the pathways of waste excretion in the brain and providing new targets for some CNS diseases.

**Figure 4:**
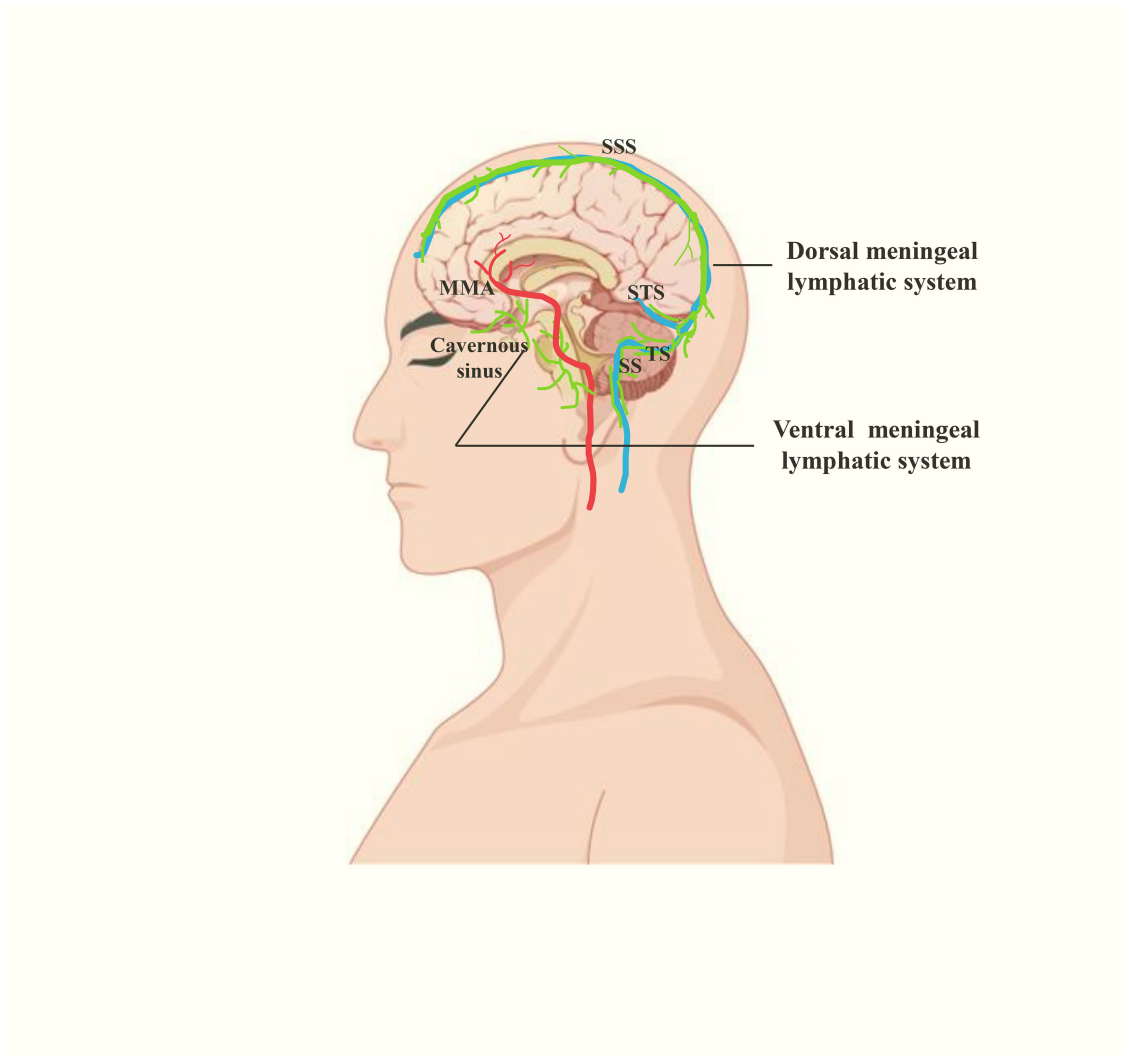
The human meningeal lymphatic system(green), including ventral and dorsal meningeal lymphatic systems. The former is located near the cavernous sinus, neural foraminas and MMA. The latter is located near the dural venous sinuses. STS, straight sinus.

Moreover, the characterization of human meningeal lymphatic flow has undergone transformative progress through advanced neuroimaging technologies. In 2017, Martina Absinta et al. first performed MRI on the meningeal lymphatic system in human and non-human primate by using a contrast agent (gadobutrol) to specifically differentiate lymphatic vessels and blood vessels, visualizing the MLVs located near the dural sinus, MMA and cribriform plate, some of which are completely enclosed within the dura (**Figure 4**) (9). In 2019, Phillip H. Kuo et al. found that the direction of lymph flow in the MLVs near the SSS is toward the cribriform plate and opposite to the direction of blood flow in the SSS (29). Jun-Hee Kim et al. proposed a noninvasive MLVs imaging technique based on an inter-slice blood perfusion MRI, called alternate ascending/descending directional navigation (ALADDIN), by which they verified the flow velocity of the dorsal MLVs in humans ranges between 2.2 and 2.7 mm/s (43). In 2020, Geir Ringstad et al. demonstrated that CSF tracers flow into the parasagittal dura in humans and Ying Zhou et al. discovered the old have the impairment of meningeal lymphatic drainage and thickening of lymphatic channels in the both dorsal and ventral regions (44, 45). Therewith in 2022, Laurent Jacob et al. demonstrated the extended anterior MLVs network around the cavernous sinus in human, with exit routes through the foramina of emissary veins, via real-time vessel-wall MRI (VW-MRI) (**Figure 4**) (46). Furthermore, Mehmet Sait Albayram et al. imaged not only intracranial MLVs but also the pathway of drainage from CNS to dCLNs. They classified the human meningeal lymphatic system into dorsal and ventral systems(**Figure 4**). The former refers to the MLVs near the dural sinus, which exits the skull through the JF and foramen magnum. The ventral system includes the MLVs located near the anterior cranial fossa, optic groove, Meckel’s caves and internal auditory canals, leaving the skull along the cranial nerves. The two eventually converge to dCLNs, and together participate in removing waste from the brain (47).

## Regulation of meningeal lymphatic drainage

As a special lymphatic vessel, MLV drainage can be regulated by various molecules or stimulations, via modulating the structure or contractile function (**Tables 1**–**3**).

**Table 1:** Molecular regulators of MLVs and effects.

Molecules	Function mechanism or post-activation effects
VEGFR-3	Increasing the diameter of MLVs; Promoting the growth and migration of LECs, which may involve CACNA1D and Decorin
Piezo1	Activation of eNOS, VEGFR2/3 and AKT1 in LECs; Affecting the expression of VE-Cadherin to alter the connection between LECs
CGRP	Affecting the expression of VE-Cadherin, Connexin-47, PTX3 and MADCAM1 in LECs, leading to the change of intercellular junctions and decreased permeability
IFN-γ	Disrupting the VE-Cadherin junctions of LECs
DSCR1	Helping to maintain the structural and functional integrity of MLVs, which may involve Wnt signalling pathways

**Table 2:** Non-invasive modulation of meningeal lymphatic drainage.

Regulation mode	Effects
rTMS	Enhancing the glymphatic function and meningeal lymphatic drainage
Near-infrared light irradiation	Ameliorating the mitochondrial metabolism and junctions of LECs to enhance the meningeal lymphatic drainage
40 Hz stimulation	Promoting the function of the glymphatic system and meningeal lymphatic drainage
Physical exercise	Increasing the size and flow of MLVs and glymphatic influx
Visudyne	Damaging LECs and resulting in MLV injury after photoconversion

**Table 3:** Modulation of extracranial lymphatics.

Regulation mode	Effects
rTMS	Enhancing the glymphatic function and meningeal lymphatic drainage
Near-infrared light irradiation	Ameliorating the mitochondrial metabolism and junctions of LECs to enhance the meningeal lymphatic drainage
40 Hz stimulation	Promoting the function of the glymphatic system and meningeal lymphatic drainage
Physical exercise	Increasing the size and flow of MLVs and glymphatic influx
Visudyne	Damaging LECs and resulting in MLV injury after photoconversion

### Molecular regulators of meningeal lymphatic function

#### VEGFR-3

The vascular endothelial growth factor-C/D (VEGF-C/D) signaling pathway conducted by vascular endothelial growth factor receptor-3 (VEGFR-3) plays a significant role in lymphangiogenesis and development (48–51). K14-VEGFR-3-Ig transgenic mouse, which has an impaired VEGF-C/D–VEGFR3 pathway, exhibits disabled MLVs and significantly weakened function in draining macromolecules from the brain to dCLNs (6, 52). Expressing the extracellular domains 1 to 3 of murine VEGFR-3 by adeno-associated virus (AAV), which bind and sequester VEGF-C/D (VEGF-C/D trap), can induce meningeal lymphatic degeneration and drainage dysfunction (53). Administering a monoclonal antibody against VEGFR-3 or specifically knocking out VEGFR-3 in adult mouse LECs yields similar results (54). Conversely, intracranial overexpression of VEGF-C in aged mice through AAV, osmotic drug delivery from the skull through hydrogel, direct injection into cisterna magna, or delivery of VEGF-C mRNA all can increase the diameter of MLVs and enhance the drainage function (10, 55–58). The mechanism involves promoting the growth of LECs and changes in transcriptomics, including the increased calcium voltage-gated channel subunit α1 D (Cacna1D) expression and decreased Decorin expression, which are related to the proliferate and migrate of LECs (58). Moreover, skull progenitor cells secrete VEGF-C, which promotes the growth and migration of LECs and facilitates the growth and function of MLVs, playing an important role in craniosynostosis (59).

#### Piezo1

Piezo1 is a mechanically gated cation channel that senses the mechanical forces applied to the cell membrane and cytoskeleton (60, 61), playing a crucial part in the development and function of lymphatic vessels (62–64). Yoda1, a small molecule chemical agonist of Piezo1, lowers the mechanical force threshold for activation (65). Administering Yoda1 to mice stimulates MLVs to drain CSF. The mechanism may involve the activation of endothelial nitric oxide synthase (eNOS), VEGFR-2/3 and protein kinase B1 (AKT1) in LECs. Moreover, Yoda1 affects the expression of VE-Cadherin to alter the connection between LECs (66, 67). Specifically knocking out or overexpressing Piezo1 in the lymphatic vessels of mice also results in decreased or enhanced meningeal lymphatic drainage. Notably, knocking out or overexpressing Piezo1 for a short period does not cause changes in the structure of the MLVs, but the drainage function still appears weakened or enhanced (67).

#### CGRP

Calcitonin gene related peptide (CGRP) is related to sympathetic nerve excitation transmission and has strong vasodilator activity (68, 69). However, LECs even have higher expression levels of the CGRP receptor (a dimer formed by calcitonin receptor-like receptor, CLR and receptor activity modifying protein 1, RAMP1) than vascular endothelial cells (70, 71), and the abnormal expression of both will affect the development and function of lymphatic vessels (72, 73). At the cellular level, CGRP affects the VEGFR-3 signaling pathway and also alters the expression of some intercellular connection-related proteins such as VE-Cadherin (74, 75). In MLVs, CGRP stimulation reduces their permeability and inhibits their drainage function. The mechanism involves affecting the expression of VE-Cadherin, Connexin-47, Pentraxin3 (PTX3) and mucosal vascular addressin cell adhesion molecule 1 (MADCAM1) in meningeal LECs, leading to the changes of the connections between endothelial cells (76).

#### Interferon-γ

Interferon-γ (IFN-γ), a cytokine with roles in antiviral and anti-tumor responses, can be secreted by T cells, which can accumulate in the dura mater especially during the ageing (11, 77). A study conducted by Justin Rustenhoven discovered that the level of IFN-γ in meningeal LECs is increased in aged mice and the overexpression of IFN-γ disrupted VE-Cadherin junctions of LECs, impairing the meningeal lymphatic drainage (78). Manipulating the level of IFN-γ by intraperitoneally injecting anti-IFN-γ neutralizing antibodies can improve the flow of MLVs in aged mice, which may function by reducing the damage to VE-Cadherin caused by IFN-γ (78). Noteworthily, they administered peripherally but the lymphatic flow in CNS got significant improvement, highlighting its potential for clinical translation.

#### DSCR1

Down syndrome critical region 1 (DSCR1, also known as regulator of calcineurin 1, RCAN1) is upregulated in the tissues of patients with Down syndrome (79), especially in the brain, mechanistically linking to learning and memory impairments (80). However, in LECs, VEGF mediates the induction of DSCR1 (81), suggesting its potential role as a downstream effector in VEGF-driven lymphangiogenesis. Chiyeol Choi et al. found overexpressing DSCR1 potentiated dorsal MLVs and augmented intracranial lymphatic drainage capacity (82). In 5×FAD mice, increasing DSCR1 rescued the structural and functional integrity of MLVs and assisted to the discharge of Aβ, ultimately ameliorating cognitive deficits (82). Transcriptomic profiling of meningeal LECs implicated Wnt signaling pathways functioning in this reparative process, although the precise molecular mediators responsible for meningeal lymphangiogenesis remain to be elucidated.

### Non-invasive modulation of meningeal lymphatic drainage

For its convenience, non-invasive stimulation has been extensively studied as a promising therapeutic target for various diseases, including some neurodegenerative diseases, such as AD and PD (83–85). Additionally, research has explored its effects on MLVs. Common stimulations include light, sound and magnetic stimulation. Yangyang Lin et al. found that repetitive transcranial magnetic stimulation (rTMS) promotes meningeal lymphatic drainage and glymphatic function in 5×FAD mice, reducing the Aβ deposition in the brain (86). Miao Wang and Dongyu Li et al. used near-infrared light irradiation and found enhanced drainage function in MLVs (87, 88). By transmission electron microscopy imaging and RNA sequencing, they found that light modulation ameliorated the mitochondrial metabolism and junctions of meningeal LECs, and subsequently enhanced the capability of MLVs in old and AD mice. In addition, Mitchell H. Murdock et al. and Wen Wu et al. respectively reported that multisensory 40 Hz stimulation (combining light and sound) or 40 Hz blue light exposure enhances the efflux of Aβ to dCLNs in AD mice, although this process is indirectly achieved by promoting the function of the glymphatic system, rather than directly acting on MLVs (41, 89).

Besides, regular voluntary exercise has been shown to increase the waste discharge through glymphatic system in mice brain (90). In healthy volunteers, Roh-Eul Yoo et al. investigated the impact of physical exercise on brain waste clearance via MRI (91). The results showed the size and flow of MLVs increased significantly after long-term exercise, which was associated with the downregulation of S100 calcium-binding protein A8 (S100A8), S100A9, proteasome subunit alpha type-3 (PSMA3), and defensin alpha 1 and alpha 3 (DEFA1A3) and the up-regulation of J chain in plasma (91).

In addition, to experimentally damage MLVs, researchers use a photodynamic drug named visudyne to ablate MLVs, which preferentially damages LECs after photoconversion (92). They inject visudyne into CSF and proceed photoconversion, resulting in MLV injury and loss of drainage function (4, 10, 55). Notably, repeating the above operations can prolong the effect of ablation (10), providing a model of long-term MLVs damage.

### Modulation of extracranial lymphatics and clinical translation

As a component of the pathway from MLVs to dCLNs, dCLVs also play an important part in CSF drainage. Due to the existence of smooth muscle in dCLVs, some drugs that dilate or constrict blood vessels can also be applied in dCLVs. Phenylephrine can activate α1-adrenergic receptors in lymphatic smooth muscle cells and contract dCLVs, reducing the CSF drainage (31). By releasing nitric oxide (NO) and increasing the intracellular level of cyclic guanosine monophosphate (cGMP), sodium nitroprusside can act on smooth muscle cells and dilate dCLVs, enhancing the CSF outflow (31, 93). It is worth noting that a low concentration of phenylephrine can also dilate dCLVs and promote CSF drainage, probably through β2-adrenergic receptors (31). However, a study conducted by Ting Du et al. reported that the reduction of contraction frequency and flow velocity occurred in aged CLVs and slowed the CSF outflow (94). Topical application of prostaglandin F2α, a prostanoid that increases smooth muscle contractility, could restore lymphatic function and CNS clearance (94).

Apart from pharmacological methods, surgical ligation of the afferent or efferent lymphatic vessels of dCLNs obstructs CSF drainage by MLVs (6, 10, 55, 95, 96). Some researchers even directly bridged the subdural space above the hippocampus with the submandibular lymph node to guide the lymphagiogenesis of MLVs (97). Targeting downstream components of the meningeal lymphatic axis represents a translational strategy to augment cerebral waste clearance without direct CNS intervention.

Recently, an operation named deep cervical lymphatic-venous anastomosis (dcLVA) has been performed on AD patients and reported to improve cognition (98). Surgeons connect dCLVs marked by ovalbumin with indocyanine green (ICG) with jugular veins, to enhance intracranial lymphatic drainage and waste removal in CSF, then evaluate curative effect by MRI and cognitive restoration (98). Besides, some similar surgical methods like lymph node-venous anastomosis (LNVA), lymphoid tissue-venous anastomosis have been also used for the treatment of AD (99). However, the feasibility still needs more clinical data to confirm, and long-term patency rates of surgical anastomoses remain undetermined (long-term follow-up data lacking). Moreover, with the same theory, we can even propose a bold hypothesis: does CSF shunt surgery like ventriculo-peritoneal shunt has a similar curative effect? Overall, dCLVs have their own advantages. Locating outside the CNS, dCLVs can be manipulated more easily to affect CSF drainage compared with MLVs, presenting a promising target for brain waste removal.

## MLVs in brain ageing and CNS diseases

### Brain ageing

**Figure 5:**
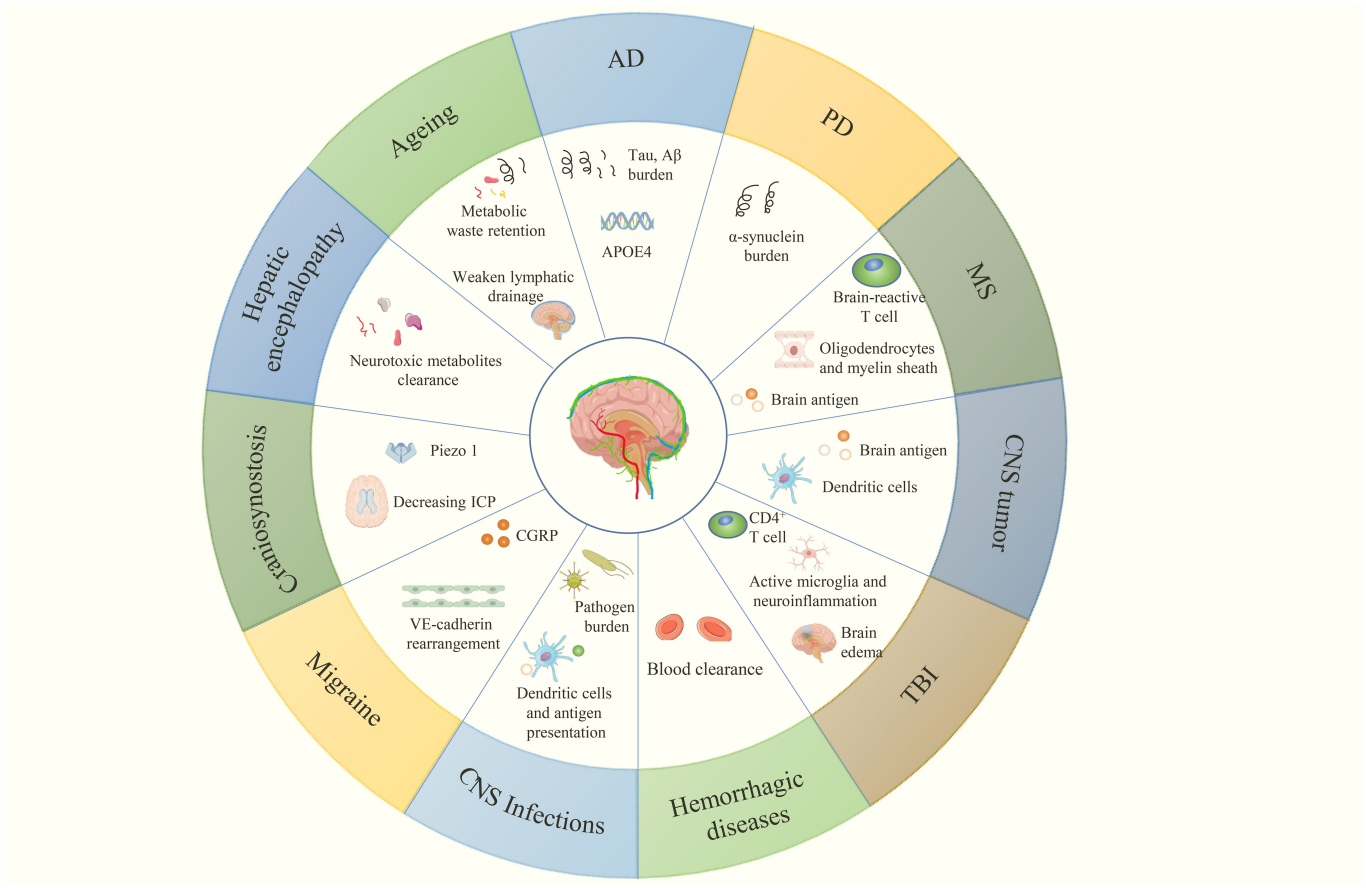
CNS diseases related to MLVs and their multiple aspects regulated by meningeal lymphatic (dys)function, which are discussed in this review.

Brain ageing is intricately linked to the deterioration of physical and mental health (100). Ageing triggers the changes in brain structure, such as reductions in regional cortical thickness, subcortical volume, rarefied white matter, and enlargement of the ventricles, and the worsening of this process contributes to the pathogenesis of neurodegenerative disorders, such as AD and PD. In aged mice, both the structural integrity and function of MLVs are impaired (10, 18, 30), while the deterioration of glymphatic function also occurs and is associated with the deficit of C-C chemokine receptor type 7 (CCR7) on meningeal T cells (101). These changes jointly lead to the weakening of meningeal lymphatic drainage, participating in causing some behavioral changes like suppression of exploratory activity and cognition (13, 101). As the main carrier of CSF drainage, basal MLVs show increasing in size, high branching and hyperplastic phenotypes but have fewer valves and impaired LEC junctions, ultimately leading to an attenuated drainage function (18). Noteworthily, giving VEGF-C or non-invasive modulations like near-infrared light treatment can delay the ageing of MLVs and restore their function (10, 87). Similar changes occur in aged humans. MRI results show that elderly individuals have worse meningeal lymphatic drainage and thickened MLVs (45, 47), causing metabolic waste retention in the brain, which may aggravate ageing in turn (**Figure 5**).

### AD

The pathogenesis of AD is closely related to the abnormal deposition of Aβ and neurofibrillary tangles formed by hyperphosphorylated tau in the brain (102). MLVs play a significant part in clearing these harmful molecules.

Interestingly, Aβ was initially isolated from the meningeal tissue of AD patients (103). Indeed, MLVs can transport Aβ from the brain to dCLNs. Disruption of meningeal lymphatic drainage(e.g., photodynamic ablating the MLVs, deficit in CCR7 or ligating the dCLVs) leads to Aβ deposition (**Figure 5**), exacerbated brain pathology, and decline in cognize and memory in AD transgenic mice including J20, 5×FAD and APP/PS1 (10, 96, 101, 104). Conversely, improving meningeal lymphatic drainage alleviates Aβ deposition. Giving VEGF-C to (old) AD transgenic mice in different ways improves MLVs function and promotes Aβ efflux to dCLNs (10, 56, 104). Non-invasive stimulations like near-infrared light irradiation and rTMS yield similar results in 5×FAD and APP/PS1 mice (86–88). Noteworthily, according to a study published in January 2025, cranial bone maneuver (CBM) promotes the lymphangiogenesis and drainage of MLVs, alleviating the Aβ deposition and memory deficits in 5×FAD mice for a long time (105). Besides, some traditional Chinese medicines (TCMs) have also shown promise in promoting meningeal lymphatic flow in AD. A study in 2023 revealed that borneol decreases the NO levels to constrict MLVs and increases VEGF-C and LYVE-1 to stimulate the lymphangiogenesis and valve plasticity, thereby contributing to Aβ efflux (106). A formula named Jiawei Xionggui decoction has also been proven to improve the Aβ efflux via MLVs and neuroinflammation by inhibiting the arachidonic acid pathway (107). However, in these studies about TMCs, the sample size limits the clarification of the problem. Only 3 to 5 samples were included in each group when studying the structure and drainage function of MLVs. Besides, all the above-mentioned treatment methods have been proved effective in the AD model mice, but whether they have same efficacy and safety in human remains to be verified.

Equally, tau can also be exported to the periphery by MLVs (108). Injecting labelled tau into the brains of K14-VEGFR-3-Ig transgenic mice results in more signal retention in the brain compared with mice having normal MLVs function, suggesting that MLVs regulate the clearance of extracellular tau from the CNS (52). Of course, without the action of glymphatic system, tau cannot get out from the brain parenchyma to CSF and enhancing glymphatic system facilitates the meningeal lymphatic clearance of tau in parenchyma (40). Interestingly, Mingqi Liu and Shiying Dong et al. found Interleukin 33(IL-33) and cannabidiol can enhance glymphatic function and intracranial lymphatic clearance, reducing tau accumulation and ameliorating memory and cognitive functions in the model of TBI (109, 110).

Moreover, apolipoprotein E4 (APOE4), the leading genetic risk factor for AD, may be associated with MLVs (**Figure 5**) (102, 111, 112). Although not yet proven, the connection between APOE4 and peripheral lymphatic (113) are always driving scientists to research its role in MLVs (111). By reanalyzing the previously published RNA-Seq data, Alexios-Fotios A. Mentis et al. proposed that induced pluripotent stem cells (iPSCs) carrying the APOE4 allele show lower expression of genes related to lymphatic markers and valve formation. Consequently, they put forward that APOE4 may induce abnormalities in the structure and function of MLVs (lymphosclerosis and lymphedema), then impair the clearance of waste like Aβ and exacerbate the manifestations of AD (111), although the authenticity of this conjecture has not yet been verified.

### PD

PD is a neurodegenerative disease characterized by the loss of dopaminergic neurons in the substantia nigra and the formation of Lewy bodies, in which α-synuclein plays an essential role (114, 115). MLVs can drain oligomeric α-synuclein and reduce macrophage activation (116). Ligating the dCLVs of A53T mice that express mutated human α-synuclein aggravates α-synuclein accumulation (**Figure 5**), inflammation and dopaminergic neuronal loss (95). After injecting α-synuclein preformed fibrils into the brains of mice more than 6 months, Xue-Bing Ding et al. observed that the delayed clearance of CSF and loss of the tight connections between meningeal LECs, suggesting that α-synuclein may injure MLVs in PD (117). Besides, they also found that patients with idiopathic PD exhibit significantly reduced flow in the MLVs along the SSS and SS, as well as a delay in dCLNs perfusion, first demonstrating the relationship between PD and MLVs in human (117).

### MS

MS is an autoimmune disease of CNS, characterized by mistaken immune system attack and demyelination of CNS (118). As components related to the immune system, Antoine Louveau et al. proposed that MLVs might participate in MS at the outset of discovery (14). In 2019, they found MLVs transport the brain antigens to T cells in dCLNs, causing T cells to recognize brain antigens and attack the CNS components (**Figure 5**) (4), indicating that MLVs do not always have a positive effect on CNS diseases. Ablating the MLVs or ligating the afferent lymphatic vessels of dCLNs relieves pathology (4). However, meningeal lymphatics also regulate oligodendrocyte function and brain myelination, and meningeal lymphatic dysfunction hinders brain myelination (53). Furthermore, MLVs did not have morphological changes (4), whereas the NPLP near the cribriform plate proliferated and drained both CSF and cells previously in the CNS parenchyma in experimental autoimmune encephalomyelitis (EAE) mice(a MS model) (119, 120), suggesting that NPLP may also play a part in neuroinflammation. At present, the main treatment method for MS in clinical practice is disease-modifying therapies (DMTs). Most DMTs have anti-inflammatory effects and function by inhibiting peripheral immunity and the entry of peripheral lymphocytes into CNS, which may lead to adverse consequences such as an increased risk of infection (121). Therefore, blocking the transport of brain antigens to the periphery is promising in the treatment of MS. How to block the brain antigens transport by MLVs without affecting oligodendrocytes may be the next research hotspot.

In 2025, a research conducted by Min Woo Kim et al. put forward that there are endogenous self-peptides existing in the brain’s borders and its lymphatic drainage pathway, which guide the immune response towards suppression, whereas neuroinflammation can impair the presentation of these peptides and worsen the immune response (122). Replenishment of these peptides in CSF ameliorates EAE, providing a new direction for the treatment of CNS autoimmune disease (122).

### Tumors

As mentioned above, MLVs can export cells and antigens in the brain to dCLNs and present them to T cells (4, 123), which also function in CNS tumors. Glioma, a common CNS tumor, has always been a research hotspot. Qiaoli Ma et al. reported that mice with gliomas showed decrease of CSF outflow and lower signals in dCLNs after injecting CSF tracer (124). The glioma patients and mice both have low expression of VEGF-C, and administering VEGF-C to these mice prolongs their survival while ligating the dCLVs cancels this effect (57). In 2020, Xueting Hu et al. found that dorsal MLVs remodeling happened in mice with intracranial glioma or metastatic melanoma. And mice with defective or enhanced dorsal MLVs transported fewer or more dendritic cells (DCs, carrying the antigens in the brain) from CNS tumors to dCLNs (**Figure 5**) (125). Besides, combining checkpoint blockade therapies with VEGF-C delivery improves the therapeutic effect on glioma (57, 125). In 2022, Changping Zhou et al. reported that delivering VEGF-C mRNA significantly enhanced radiotherapy efficacy by MLVs in mice with brain tumors (126). Notably, VEGF-C shows low expression in patients with glioma and the level is strongly correlated with the anti-tumor immune response (57, 126). It is of great significance to conduct VEGF-C-related clinical trials on patients with glioma. In 2023, Minghuan Wang et al. found that patients with CNS malignant tumors presented elevated wash-in function and decreased wash-out function of MLVs, which represent the ability of MLVs to absorb waste from brain and expel it (127). These phenomena are associated with degree of tumors malignancy, isocitrate dehydrogenase (IDH) genotype and disease progression (127), but how these factors affect MLVs and whether MLVs are associated with CNS malignant tumor prognosis still need to be explored.

### TBI

Studies in recent years have shown correlations between MLVs and the progression and recovery of TBI (109, 110, 128–133). In terms of gene expression, meningeal LECs of TBI mice show lower LYVE-1 expression but higher expression of FMS-like tyrosine kinase 4 and neuropilin 2 (markers of lymphangiogenesis), suggesting MLVs may function in the recovery of TBI (132). Indeed, TBI causes severe deficits in meningeal lymphatic drainage and increases intracranial pressure (ICP), while a high ICP injures MLVs, leading to a vicious cycle (130). In addition, K14-VEGFR-3-Ig mice show a significant reduction of infiltrating CD4^+^ T cells in the brain after TBI, suggesting that MLVs may participate in the neuro-immune response (**Figure 5**) (133). The mechanism of meningeal lymphatic drainage impairing after TBI mainly involves the “adrenaline storm” theory (129). Rashad Hussain et al. proved that the excessive systemic release of noradrenaline after TBI would cause a suppression of glymphatic fluid flow and reduced contractility of dCLVs, leading to the damage of meningeal lymphatic drainage. Pan-adrenergic receptor inhibition partly restored intracranial lymphatic flow and promoted the export of cellular debris, improving the brain edema and prognosis (129). Besides, pre-injured MLVs worsen the inflammation caused by TBI, which may be related to the upregulated expression of complement-related genes and the activation of microglia (128, 130). These findings suggest that the old with weaker MLV function may get more severe consequences after TBI. Some drugs that can improve the meningeal lymphatic drainage have been proven to have therapeutic effects in TBI mice. VEGF-C, ketoprofen and 9-cis retinoic acid improve the neuroinflammation and brain edema caused by TBI through stimulating MLVs to express LYVE-1, VEGFR-3, PROX-1 and forkhead box C2 (FOXC2) and enhancing drainage (130, 131). IL-33 and cannabidiol are also beneficial for the recovery of TBI by facilitating the lymphatic drainage of toxic waste (109, 110). They improve the exchange of CSF and ISF by reversing the dysregulation and depolarization of AQP-4. In other words, they can enhance the glymphatic system. Notably, these interventions can also inhibit the activation of glial cells and improve neuroinflammation, providing new methods for the reconstruction of neurological deficits in TBI patients (109, 110, 130, 131). Although the efficacy and safety (or side effects) in human still need to be verified through clinical trials.

### Hemorrhagic diseases

Hemorrhagic diseases, including subarachnoid hemorrhage (SAH), intracerebral hemorrhage (ICH) and subdural hematoma (SDH), pose a serious threat to CNS.

The subarachnoid space is filled with CSF and MLVs can drain the CSF to periphery, so it is easy to consider MLVs when researching SAH. Indeed, in 2019, Tinglin Pu et al. found that the drainage function of MLVs decreased within one week after SAH in mice (134). Similarly, in larger animals like dogs, SAH can also disrupt MLVs (135). In 2020, Jinman Chen et al. demonstrated that MLVs drain the blood in subarachnoid space to dCLNs in SAH mice (**Figure 5**) (136). Additionally, they found that ablating MLVs or inhibiting VEGFR-3 reduces the clearance of the blood in subarachnoid space and aggravates the pathology of SAH (136). Subsequently, by single-cell RNA sequencing and spatial transcriptomics, Xiaoyu Wang et al. revealed that SAH induces MLV injury and the outcome of SAH is associated with thrombospondin 1 (THBS1) and S100A6. Furthermore, the THBS1-CD47 ligand-receptor pair functions in LECs apoptosis via signal transducer and activator of transcription 3/B-cell lymphoma 2 (STAT3/Bcl-2) signaling (137). Also, enhancing Th17 cell drainage through MLVs could alleviate neuroinflammation after SAH and influence prognosis (138).

ICH can also be relieved by MLVs (139, 140). After ICH, MLVs expand and become more functional, simultaneously drain the blood to dCLNs (140). During the late phase of ICH, MLVs proliferate (139). Ablation of MLVs or ligation of dCLVs inhibits the clearance, whereas administering VEGF-C promotes. Moreover, enhanced MLVs reduce the neuron loss, glial cell activation and hematoma volume (139). A non-invasive stimulation, rTMS, can enhance the clearance of intracranial lymphatic drainage after ICH (141). Also, near infrared photobiomodulation provides fast recovery after intraventricular hemorrhage (142). A drug named Panax Notoginseng Saponins (PNS) also promotes the drainage function of MLVs by stimulating the lymphangiogenesis in ICH (143). Additionally, in ischemic stroke, VEGF-C pretreatment enhanced MLVs, leading microglia-mediated inflammation mitigating and neuroprotective factors increasing, and helping to reduce stroke injury (58). Notably, cranial bone transport (CBT) has been found to contribute to the remission of neurological deficits in ischemic stroke patients (144), in which MLVs may also provide assistance (145). Interestingly, middle cerebral artery occlusion (MCAO) animals (an ischemic stroke model) have worsen meningeal lymphatic drainage and the animals with ablation MLVs or mutational VEGFR-3 get more grievous stroke injury after MCAO, however, the stroke mice induced by photothrombosis show meningeal lymphangiogenesis (145, 146). Why different molding methods have different effects on MLVs still remains unknown. Perhaps the laser used in photothrombosis has some stimulatory effect on meningeal LECs? This could be an interesting question to explore.

Equally, the SDH shares similar characteristics. Xuanhui Liu et al. found the red cells in dCLNs of SDH model rats, and ligating dCLVs aggravated the SDH (147). After SDH, the expression of lymphangiogenesis-related proteins in meninges, including LYVE1, FOXC2 and VEGF-C, is downregulated (147). In this process, MLVs are damaged, leading to progressive functional loss. The injury of MLVs, especially the basal MLVs, may be related to the dephosphorylation of extracellular signal-regulated kinase 1/2 (ERK1/2) within the LECs and the resulting change in connections between LECs (148). Besides, the vitamin D treatment significantly reduces SDH volume and improves the drainage of SDH to cervical lymph nodes (149).

### CNS infections

The meningeal lymphatic system demonstrates functional involvement across major categories of CNS infections, encompassing bacterial, viral, and parasitic pathogens. In *Listeria monocytogenes* (LM) infection, bacterial invasion induces paradoxical responses in MLVs: lymphangiogenesis initially occurs, accompanied by the downregulation of critical lymphatic developmental regulators (FOXC2 and GATA binding protein 2, GATA2), ultimately culminating in structural degeneration and impaired drainage capacity (150). Notably, experimental ablation of MLVs exacerbates neuroinflammation and increases the bacterial burden in LM-infected mice (**Figure 5**) (150). Moreover, modulation of meningeal lymphatic function (including enhancement and inhibition) correspondingly alters outcomes in lipopolysaccharide-induced sepsis-associated encephalopathy (SAE) (151). This finding positions MLVs as a potential therapeutic target— capable of mitigating inflammation driven by infection.

Neuroinvasive viruses including Japanese encephalitis virus (JEV), rabies virus (RABV), and herpes simplex virus 1 (HSV-1), induce lymphangiectasia while impairing the physiological drainage of MLVs (55). MLVs mediate viral egress from CNS to dCLNs, and damaging or enhancing the MLVs gets similar results as the bacterial infection (55). Furthermore, as an important structure in CNS infection, a provocative hypothesis suggests MLVs may facilitate Human Immunodeficiency Virus (HIV) neuroinvasion, though the truth remains pending (152).

In parasitic infections such as *Toxoplasma gondii* invasion, MLVs orchestrate adaptive immune priming— DCs transport parasite antigens via MLVs to dCLNs, enabling peripheral T cells activation against CNS-resident pathogens (**Figure 5**) (153). VEGF-C treatment induces meningeal lymphangiogenesis and improves CSF outflow in chronically Toxoplasma gondii infected mice, although fails to alleviate infection-associated cerebral edema (154), highlighting the uncoupling of lymphatic flow and symptom improvement.

### Other CNS diseases

In addition, several other CNS diseases are also associated with MLVs. In migraine pathophysiology, elevated meningeal levels of pro-inflammatory cytokine IL-12-p70 and CGRP correlate with lymphatic impairment (155). Neuroimaging studies have revealed meningeal lymphatic flow abnormalities in chronic migraine patients (156). Mechanistically, CGRP signaling induces VE-Cadherin rearrangement in MLVs (**Figure 5**), decreasing endothelial permeability and impairing drainage capacity— a pathway corroborated by migraine symptom alleviation following CGRP receptor blockade (76).

Therapeutic modulation of meningeal lymphatic function demonstrates translational potential in structural brain disorders. In craniosynostosis models, pharmacological activation of mechanosensitive Piezo1 via Yoda1 restores CSF drainage and normalizes the ICP (**Figure 5**) (66). Transplantation of VEGF-C-secreting skull progenitor cells induces meningeal lymphangiogenesis, effectively ameliorating craniosynostosis-associated neurological deficits (59).

Furthermore, in rats subjected to bile duct ligation, elevating VEGF-C in CSF enhances meningeal lymphatic clearance of neurotoxic metabolites and mitigates neuroinflammation and cognitive impairment in hepatic encephalopathy models (**Figure 5**) (157), implicating that MLVs take part in mediating the liver-brain axis interactions.

## Imaging advances in MLVs and drainage path

Numerous innovative imaging methods have been employed in MLV research. Here, we make a summary of these methods.

### Optical imaging

In addition to immunofluorescence (IF) of lymphatic markers, including LYVE-1, Prox-1, VEGFR-3 and podoplanin, some researchers label MLVs by injecting fluorescein into the cisterna magna or transgenic method (such as QD705 or Prox1-GFP mice), then expose the skull to the fluorescence stereo zoom microscope or multiphoton/two-photon microscope and obtain 3D or living images of MLVs (4, 18, 158). Moreover, another optical imaging method, optical coherence tomography (OCT), has been applied for visualizing MLVs *in vivo (* 158). However, owing to the small size of MLVs, they believe that OCT can only image enlarged MLVs after BBB opening (158).

The dCLNs are the end points of intracranial lymphatic drainage. After injecting CSF fluorescent tracer, researchers observe the fluorescent signals of dCLNs and the pathway via histological sections or fluorescence stereo zoom microscopes (10, 18, 30, 55, 67), with which they quantify the drainage function and visualize the meningeal lymphatic flow. Pioneering work by Qiaoli Ma et al. established differential kinetics— near infrared (NIR) tracers appear in mouse dCLNs about ten minutes earlier than in saphenous veins, conclusively validating the existence of meningeal lymphatic pathway (30). Optical imaging has the advantage of high resolution in imaging vitro tissues. However, when imaging MLVs deep in the tissues *in vivo*, the penetrability of the optical signal limits its resolution.

### MRI

To achieve higher penetration depth beyond the limitations of optical imaging, researchers have turned their attention to MRI. Owing to its superior imaging depth and noninvasive nature, MRI is widely applied in exploring the structure and function of MLVs, although its resolution is much lower than that of fluorescence microscopy imaging. Martina Absinta et al. intravenously injected gadobutrol, a gadolinium-based contrast agent with a high propensity to seep out of capillaries and into lymph vessels to enhance MLVs, and then collected T2-fluid attenuated inversion recovery (FLAIR) and T1-weighted black-blood images to visualize the meningeal lymphatic system of human and nonhuman primate (9). Since then, MRI has been extensively used in many other studies of MLVs. Although there are a variety of MRI sequences can be collected, many researchers choose the T2-FLAIR for its high sensitivity to gadolinium-based contrast agents (18, 44, 47, 117, 159, 160). For instance, Xue-Bing Ding et al. used this to compare the meningeal lymphatic drainage function between the normal controls and patients with PD (117). Noteworthily, Mehmet Sait Albayram et al. used 3D-T2-FLAIR to observe MLVs through the internal signals of protein-rich lymphatic fluid rather than contrast media (47). Furthermore, real-time VW-MRI has been applied in exploring the structure of MLVs (46). In addition, time-of-flight (TOF) angiography can distinguish the direction of flow in vessels without exogenous contrast agent, by which Phillip H. Kuo et al. explored the drainage direction of MLVs near the SSS (29), while Jun-Hee Kim et al. quantified the velocity of these MLVs via ALADDIN (43).

For its excellent temporal resolution, MRI has emerged as a powerful tool for real-time tracking of CSF dynamics, enabling researchers to map extracranial drainage pathways *in vivo*. Ji Hoon Ahn et al. collected T2-weighted 2D FLAIR and T1-weighted 3D fast low angle shot (FLASH) sequences with intrathecal gadolinium and delineated the pathway from basal MLVs to dCLNs (18). Mehmet Sait Albayram et al. used 3D-T2-FLAIR method without contrast media to capture the human CSF drainage with 0.9 mm resolution (47). With minimal invasiveness and high imaging depth, MRI is suitable for exploring human meningeal lymphatic systems and holds promise for detecting relevant pathological changes. However, visualizing submillimeter lymphatic structures without contrast agents remains technically challenging (47).

### Photoacoustic imaging

To conduct high-resolution imaging of objects deep within tissues *in vivo*, researchers have developed new imaging techniques, such as photoacoustic imaging, which combines the advantages of optical resolution and acoustic penetration depth. Fei Yang et al. used the dual-contrast functional photoacoustic microscope (DCF-PAM) to image mouse MLVs and cerebral vessels (CVs) (161). Leveraging the properties of draining large molecules in CSF, they labelled MLVs with ICG and distinguished the CVs by hemoglobin, which have different photoacoustic characteristics. With this method, they obtained overall high-resolution 3D images of MLVs, as well as real-time monitored and quantified the CSF drainage *in vivo (* 161). However, photoacoustic imaging also cannot be operated without the tracers, and the tracers need to have specific photoacoustic properties. Furthermore, when photoacoustic imaging is applied on human, the safety of the tracer also needs to be taken into consideration.

## Synthesis and future perspectives

Since being rediscovered in 2015, MLVs have emerged as pivotal regulators of CNS homeostasis through two mechanistic axes: waste clearance and antigen presentation, protecting brain in terms of metabolism and immunity. Here we make a summary about previous related research on MLVs, at the same time, we enumerate some open questions in the way of explaining MLVs, which may be the next research hotspot.

The most compelling one is how MLVs drain CSF. Are the substances in the CSF actively absorbed by MLVs or freely diffused? Is there process selective in MLVs drain? What factors can restrict substances from entering MLVs? Besides, in peripheral CpLVs, anchoring filaments modulate the button junctions between LECs to control the inflow of ISF (24). However, whether anchoring filaments exist in MLVs has not been reported so far. Furthermore, several hydrodynamic parameters, such as flow, direction and velocity, have been fully discussed in peripheral lymphatics (25). However, these parameters and their mechanisms have not been fully elucidated in MLVs.

Moreover, the current research on the regulation of MLVs is still insufficient. Aside from known factors such as age and VEGF-C, do other factors, such as gender or certain hormones affect MLVs? After all, estrogen has been reported to promote the growth of peripheral lymphatic vessels by estrogen receptor-α expressed in LECs (162, 163). Besides, how to apply the treatments for MLVs in clinical practice is worth exploring. Although its feasibility and efficacy have not yet been fully demonstrated, dcLVA is a significant step in applying the theory of meningeal lymphatic drainage to clinical diseases (98, 99). In addition, the function of the drainage ISF of lymphatic vessels contribute to absorbing drugs. Coupled with the bidirectionality of meningeal lymphatic flow and the absence of BBB blocking effect, CNS administration by MLVs may be a research focus (34).

As a bridge of CNS and periphery, MLV plays a significant role in maintaining the brain homeostasis by modulating substance exchange and immune response. However, the mechanisms governing meningeal lymphatic drainage and how to apply these mechanisms on clinical patients still require further investigation. Systematic resolution of these issues will unlock the therapeutic potential of MLVs for many CNS disorders. In sum, with respect to MLVs, we are still in the way.

## Glossary

MLVs: Meningeal lymphatic vessels
CSF: Cerebrospinal fluid
dCLNs: Deep cervical lymph nodes
CNS: Central nervous system
AD: Alzheimer’s disease
PD: Parkinson’s disease
TBI: Traumatic brain injury
MS: Multiple sclerosis
BBB: Blood-brain barrier
NVUs: Neurovascular units
LYVE-1: Lymphatic endothelial hyaluronic acid receptor 1
LEC: Lymphatic endothelial cell
Prox-1: Prospero homeobox protein 1
SSS: Superior sagittal sinus
TS: Transverse sinus
PSS: Petrosquamosal sinus
SS: Sigmoid sinus
MMA: Middle meningeal artery
PPA: Pterygopalatine artery
COS: Confluence of the sinuses
CpLVs: Capillary lymphatic vessels
ClLVs: Collecting lymphatic vessels
ISF: Interstitial fluid
VE-Cadherin: Vascular endothelial-cadherin
SMF: Stylomastoid foramina
JF: Jugular foramen
PSF: Petrosquamous fissure
dCLVs: Deep cervical lymph vessels
NPLP: Nasopharyngeal lymphatic plexus
MRI: Magnetic resonance imaging
α-PLNPs: α-phospholipid nanoprobe
Aβ: Amyloid β
AQP-4: Aquaporin-4
ALADDIN: Alternate ascending/descending directional navigation
VW-MRI: Vessel-wall MRI
VEGF-C/D: Vascular endothelial growth factor-C/D
VEGFR-3: Vascular endothelial growth factor receptor-3
AAV: Adeno-associated virus
eNOS: Endothelial nitric oxide synthase
Cacna1D: calcium voltage-gated channel subunit α1 D
AKT1: Protein kinase B1
CGRP: Calcitonin gene related peptide
CLR: Calcitonin receptor-like receptor
RAMP1: Receptor activity modifying protein 1
PTX3: Pentraxin3
MADCAM1: Mucosal vascular addressin cell adhesion molecule 1
IFN-γ: Interferon-γ
DSCR1: Down syndrome critical region 1
RCAN1: Regulator of calcineurin 1
rTMS: Repetitive transcranial magnetic stimulation
PSMA3: Proteasome subunit alpha type-3
DEFA1A3: Defensin alpha 1 and alpha 3
NO: Nitric oxide
cGMP: Cyclic guanosine monophosphate
dcLVA: Deep cervical lymphatic-venous anastomosis
LNVA: Lymph node-venous anastomosis
CBM: Cranial bone maneuver
IL-33: Interleukin 33
APOE4: Apolipoprotein E4
iPSCs: Induced pluripotent stem cells
EAE: Experimental autoimmune encephalomyelitis
DCs: Dendritic cells
IDH: Isocitrate dehydrogenase
ICP: Intracranial pressure
FOXC2: Forkhead box C2
SAH: Subarachnoid hemorrhage
ICH: Intracerebral hemorrhage
SDH: Subdural hematoma
THBS1: Thrombospondin 1
S100A8: S100 calcium-binding protein A8
STAT3: Signal transducer and activator of transcription 3
Bcl-2: B-cell lymphoma 2
PNS: Panax Notoginseng Saponins
CBT: Cranial bone transport
MCAO: Middle cerebral artery occlusion
ERK1/2: Extracellular signal-regulated kinase1/2
LM: Listeria monocytogenes
GATA2: GATA binding protein 2
SAE: Sepsis-associated encephalopathy
JEA: Japanese encephalitis virus
RABV: Rabies virus
HSV-1: Herpes simplex virus 1
HIV: Human Immunodeficiency Virus
OCT: Optical coherence tomography
NIR: Near infrared
FLAIR: Fluid attenuated inversion recovery
TOF: Time-of-flight
DCF-PAM: Dual-contrast functional photoacoustic microscope
CVs: Cerebral vessels
ICG: Indocyanine green
